# Supplementary material for: Impact of Aging Microbiome on Metabolic Profile of Natural Aging Huangjiu through Machine Learning
Source: Foods. 2023 Feb 20;12(4):906. doi: 10.3390/foods12040906 (PMC9956941; doi:10.3390/foods12040906)
Supplement: Supplementary file 1 [file foods-12-00906-s001.zip › foods-2203098-supplementary.pdf]

Supplementary File

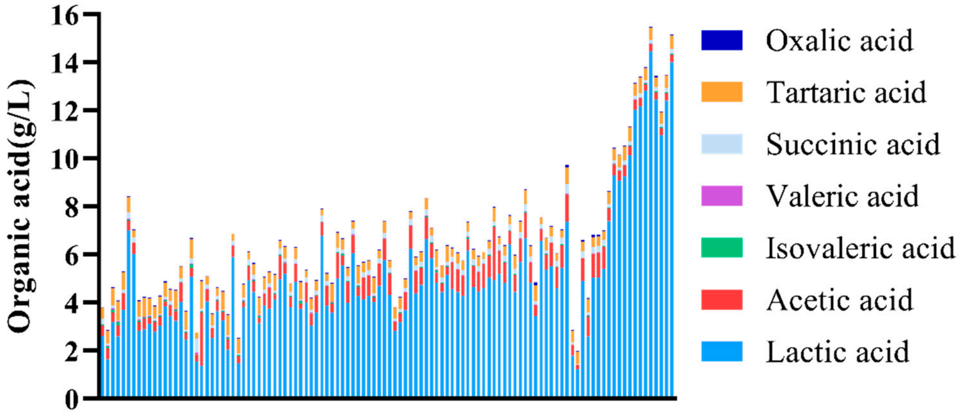

Figure S1. Contents of organic acids for aged huangjiu samples.

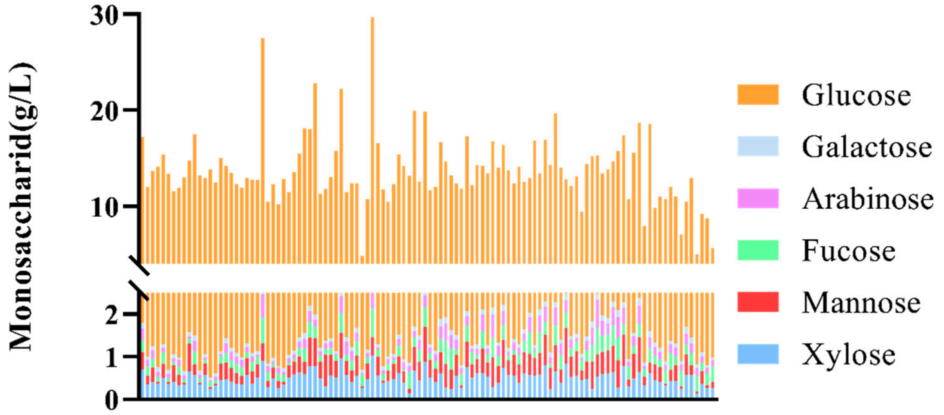

Figure S2. Contents of monosaccharides for aged huangjiu samples

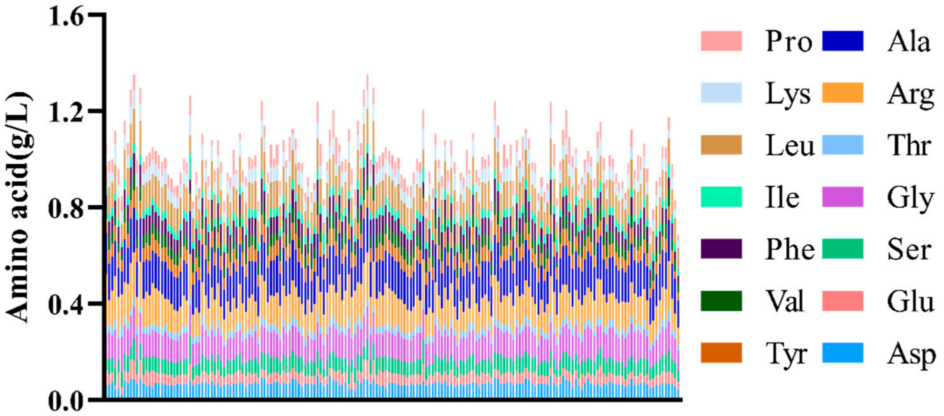

Figure S3. Contents of amino acids for aged huangjiu samples

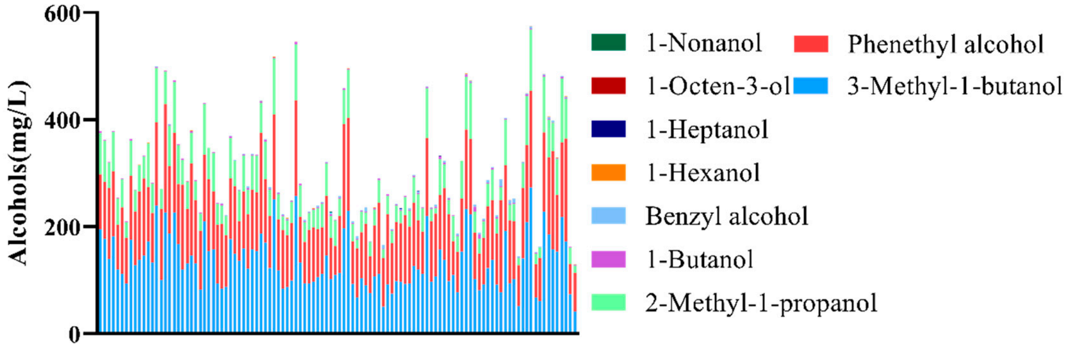

Figure S4. Contents of alcohols for aged huangjiu samples

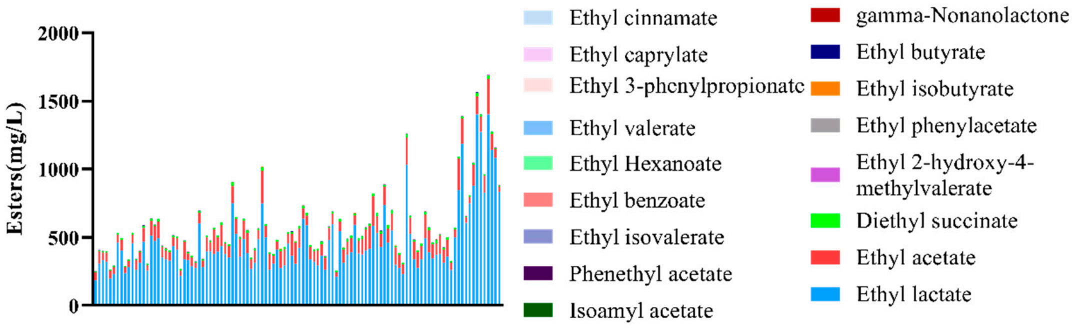

Figure S5. Contents of esters for aged huangjiu samples

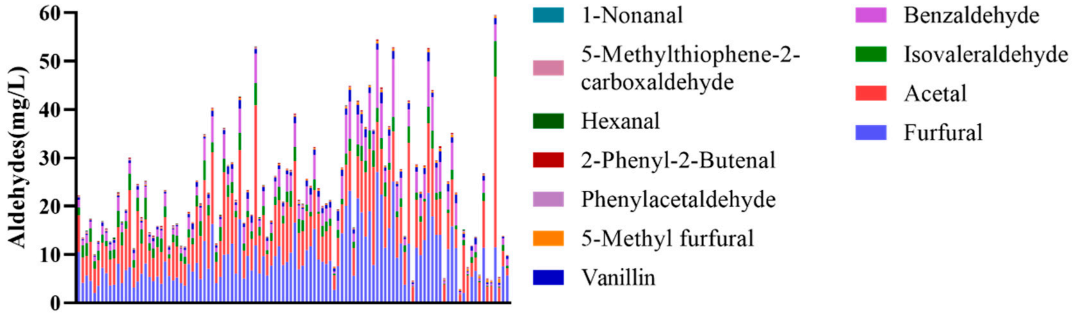

Figure S6. Contents of aldehydes for aged huangjiu samples
